# Dissolution of Metals in Different Bromide-Based Systems: Electrochemical Measurements and Spectroscopic Investigations

**DOI:** 10.3390/ma13163630

**Published:** 2020-08-17

**Authors:** Simona Varvara, Sorin-Aurel Dorneanu, Alexandru Okos, Liana Maria Muresan, Roxana Bostan, Maria Popa, Daniel Marconi, Petru Ilea

**Affiliations:** 1 Department of Cadastre, Civil Engineering and Environmental Engineering, “1 Decembrie 1918” University of Alba Iulia, 15-17 Unirii Street, 510009 Alba Iulia, Romania; roxana.bostan@uab.ro (R.B.); mpopa@uab.ro (M.P.); 2 Department of Chemical Engineering, Babes-Bolyai University, 11 Arany Janos Street, 400028 Cluj-Napoca, Romania; dorneanu@chem.ubbcluj.ro (S.-A.D.); limur@chem.ubbcluj.ro (L.M.M.); pilea@chem.ubbcluj.ro (P.I.); 3 Physical and Chemical Analysis Department (RBRO/EQV-A), SC Robert Bosch SRL, Tetarom III Industrial Park, 1, Robert Bosch Street, Jucu Herghelie, 407350 Cluj, Romania; gyorgy.okos@ubbcluj.ro; 4 Department of Molecular and Biomolecular Physics, National Institute for Research and Development of Isotopic and Molecular Technologies, 400293 Cluj-Napoca, Romania; daniel.marconi@itim-cj.ro

**Keywords:** dissolution of metals, metals recovery, bromide-based electrolytes, potentiodynamic polarization, electrochemical impedance spectroscopy, XPS, XRD

## Abstract

The dissolution of the main metals (Cu, Zn, Sn, Pb and Fe) found in waste printed circuit boards (WPCBs) was investigated by electrochemical corrosion measurements (potentiodynamic polarization and electrochemical impedance spectroscopy (EIS)) in different bromide-based systems that could be used as lixiviants in hydrometallurgical route of metals recovery. The analysis of the corrosion products was carried out by X-ray diffraction (XRD) and X-ray photoelectron spectroscopy (XPS) measurements. All measurements showed that the addition of bromine in the electrolyte favors to great extents the dissolution process of all studied metals as compared to bromine-free electrolytes. In the investigated experimental conditions, the highest dissolution rates of the metals were obtained in acidic KBr solution containing 0.01 mol/L bromine and they decreased in the following order: Zn >> Sn > Pb > Fe > Cu. The XRD and XPS chemical assessment allowed the identification of the dissolution products formed on the metallic surfaces after exposure to the electrolytes. They consisted mainly of oxides in the case of Cu, Zn, Sn and Fe, while the presence of PbBr_2_ was also noticed on the lead surface. Based on the results of EIS and surface investigations, several models explaining the corrosion behavior of the metals were proposed and discussed. The obtained results demonstrate that all studied metals could be successfully leached using brominated solutions, providing a viable alternative for the selective and efficient recovery of the base metals from WPCBs through a multi-step hydrometallurgical processing route.

## 1. Introduction

The hydrometallurgical route is a selective way toward metal recovery from waste printed circuit boards (WPCBs), being easier to control and creating less environmental hazards than the pyrometallurgical approach [1]. Various leaching reagents were used to ensure a fast kinetics of the dissolution process of both noble and base metals: cyanide [2], thiosulfate [3,4], thiourea [5,6], H_2_SO_4_ (alone or in combination with H_2_O_2_, nitric acid or other oxidants) [7,8], potassium persulfate [9], etc. Much less often, halides-based systems such as I^−^/I_2_ [10,11,12], I_2_-H_2_O_2_ [13] and Cl^−^/Cl_2_ [14] were used. To the best of our knowledge, Br^−^/Br_2_ system was used before only for extraction of gold from gold ores [15]. Fast leaching rate, low-toxicity and applicability over a wide range of pH values (from acidic to neutral) are important characteristics of the bromide leaching system [16]. On the other side, because bromine is an efficient lixiviant, but because elemental bromine is hazardous and extremely difficult to store or transport in safe conditions, it is preferable to be produced in-situ, in the leaching reactor [15,17].

In a previous research [18], we investigated the ability of the Br^−^/Br_2_ leaching system to remove the exposed metallic parts from different models of computer motherboards (CMB), simultaneous with the electrochemical lixiviants regeneration and the partial electrodeposition of the dissolved metals. Although the operating parameters were not optimized, the results indicate that CMB could be completely dismantled in about 18 h, with cathodic and anodic mean current efficiencies of 43.6% and 58.4%, respectively. Taking into consideration that leaching plays a key role in the electrochemical recycling of WPCBs, a better understanding of the metals’ dissolution mechanisms in different lixiviants might be an important step in the processes’ optimization.

In the present work, a fundamental study on the electrochemical dissolution behavior of the main base metals (Cu, Zn, Sn, Pb and Fe) found in WPCBs was carried out by corrosion measurements (potentiodynamic polarization and electrochemical impedance spectroscopy) in different bromide-based systems that could be used as lixiviants in hydrometallurgical route of metals recovery. The paper aims at assessing the electrochemical dissolution rates of the metals and establishing their dissolution mechanisms in different bromide-based electrolytes.

To gain a deeper insight into the dissolution behavior of the investigated metals, the electrochemical results were corroborated with XRD and XPS analysis of the metallic surface, after the attack of the bromide-based lixiviants. These techniques allowed the identification of the surface compounds formed during the dissolution of the metals, which might validate the corrosion mechanisms proposed based on EIS measurements. This study is part of a more complex research project aiming to elaborate an innovative and pollutants-free electrohydrometallurgical technology for metals recovery from WPCBs.

## 2. Materials and Methods

### 2.1. Materials

The working electrodes were cylindrical probes made of different pure metals embedded in epoxy resin (EpoxycureTM, Buhler, Uzwil, Switzerland): Cu (0.28 cm^2^), Zn (0.27 cm^2^), Sn (0.28 cm^2^), Pb (0.27 cm^2^) and Fe (0.28 cm^2^). Note that the working electrodes were made of pure metals instead of waste PCBs materials, and therefore they should be defined as model electrodes, simulating the metals from circuits. The exposed metallic surfaces were prepared via an abrading procedure, using successive grade of silicon carbide paper grit (from 1200 to 4000), washed thoroughly with distilled water and ethanol.

Four different bromide-based electrolytes were used as corrosive solutions:

- Sol. A: 2 M KBr (pH = 6);
- Sol. B: 2 M KBr + 0.5 M HBr (pH = 0.3);
- Sol. C: 2 M KBr + 0.5 M HBr (pH = 0.3) + 0.01 M Br_2_; and
- Sol. D: 2 M KBr + 0.5 M HBr (pH = 0.3) + 0.001 M Br_2_.

The solutions were prepared using analytical grade reagents (Sigma Aldrich, Taufkirchen, Germany and Merck, Darmstadt, Germany) and distillated water.

### 2.2. Electrochemical Measurements

A three-electrode cell was used for the electrochemical experiments. The potentials were measured against Ag/AgCl/KCl_sat_ as a reference electrode (Ref), while a twisted platinum wire (Φ = 0.5 mm, L = 10 cm) was used as counter-electrode.

The electrochemical measurements were carried out using a Princeton Applied Research (PAR) model 2273 potentiostat (Princeton Applied Research, Oak Ridge, TN, USA, 2008). Before each measurement, the working electrodes were left for at least 1 h in the corrosive solution to obtain stable open circuit potentials (OCP). In each experiment, a fresh working electrode was used, and two measurements were performed to ensure the data reproducibility.

Potentiodynamic polarization curves were recorded at a constant sweep rate of 10 mV min^−1^, in a wide potential range of ±250 mV vs. OCP, from the cathodic to the anodic direction.

Impedance spectra were recorded at OCP, in the frequency range of 100 kHz–10 MHz, at 5 points per hertz decade, using an alternating voltage amplitude of ±10 mV. The experimental impedance data were modeled using *ZSimpWin 3.21* software (EChem Software, MI, USA).

The electrochemical measurements were performed at room temperature, in the electrolytes under non-stirred and naturally aerated conditions.

### 2.3. X-ray Photoelectron Spectroscopy Measurements

X-ray photoelectron spectroscopy (XPS) data were collected using a custom built Thermofisher ESCALAB 250 Xi XPS spectrometer (Thermofisher, East Grinstead, UK) equipped with a scanning electron microscope and an X-ray detector for the acquisition of EDX data. XPS spectra were acquired using the Al Kα radiation (1486.6 eV). The data were collected from a circular surface with a radius of 650 µm. The sample’s surface was kept perpendicular to the analyzer axis for all experiments. The equipment was calibrated in the binding energy (B.E.) scale using an Au sample with the B.E. of the main Au peak observed at 84 eV. The C1s peak attributed to adventitious C was also used as a B.E. reference point for each sample. The B.E. for this peak was observed to vary between 284.6 and 285.1 eV between the samples. Survey scans were acquired in the constant analyzer energy mode (CAE) using a pass energy of 200 eV. High resolution narrow scans spectra were also recorded in the CAE mode using a pass energy of 15 eV. Depth profile experiments were performed using the lowest possible ion beam energy (typically 500 eV) to minimize the reduction of the chemical species. The XPS spectra were recorded and fitted using *Avantage* software (version 5.9915.0.6619, Thermofisher, East Grinstead, UK). The background was modeled by a “Smart” function type for all samples. The peak shape function was described by the product between a Lorentzian function and a Gaussian function with a mixing parameter of 30%. The mixing parameter was usually constrained to remain fixed.

XPS data were collected after different immersion times of the investigated metals in Sol. C, i.e., 60 min in the case of Cu, Sn and Zn and 120 min for Fe and Pb.

### 2.4. X-ray Diffraction Measurements

X-ray diffraction measurements (XRD) were carried out at room temperature on a Bruker D8 Advance powder diffractometer (Bruker, Karlsruhe, Germany) using Cu Kα_1_ radiation (λ = 0.154056 nm) at 45 kV and 40 mA. The θ–2θ Bragg–Brentano configuration geometry and incident-beam Ge (111) monochromator were used to investigate the structural properties of the samples. The 2θ range of 10°–85° was recorded at the rate of 0.02° and 2θ/0.5 s. XRD has a detection limit of ~5% by volume, if a compound is present below this level, no peaks above noise will be detected in the diffraction pattern. The exact detection limit depends on the density, Z number and crystal structure of the compounds in the sample. The crystal phases were identified comparing the 2θ values and intensities of reflections on X-ray diffractograms with JCP data base using a Diffraction AT-Brucker program.

Given the limitations of our structural detection, XRD diffractograms were collected after 120 h immersion of Cu, Zn, Sn, Pb and Fe electrodes in Sol. A, Sol. B and Sol. C, respectively.

## 3. Results and Discussions

### 3.1. Potentiodynamic Polarization Measurements

**Figure 1:**
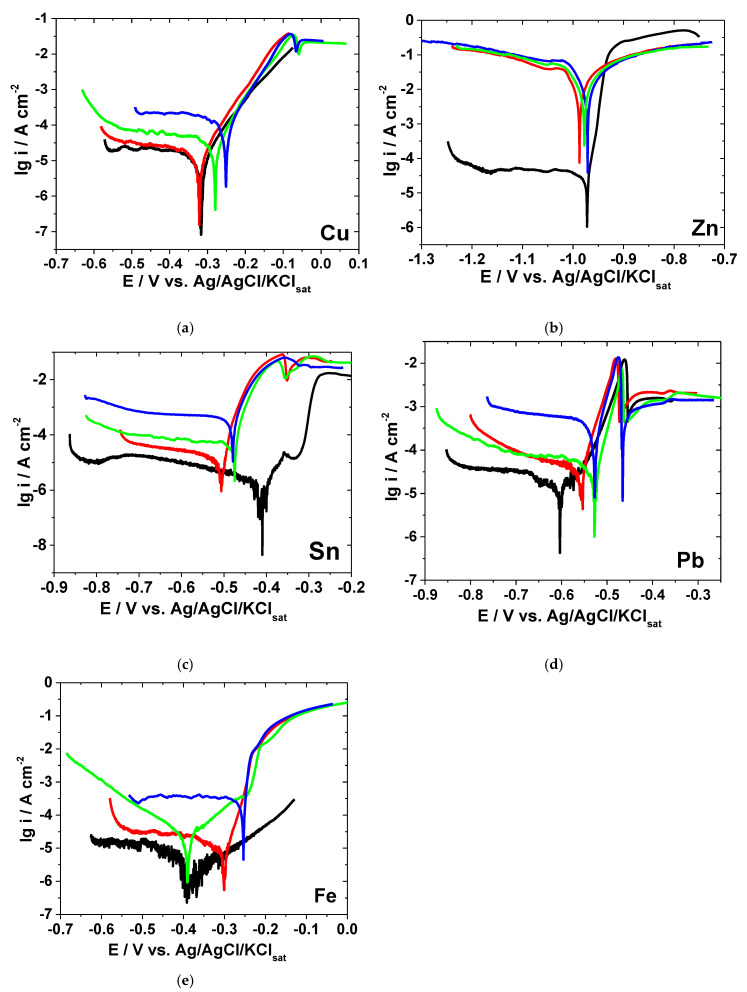
Polarization curves collected after 1-h immersion of electrodes in different bromide-based electrolytes: Cu (**a**); Zn (**b**); Sn (**c**); Pb (**d**); and Fe (**e**). (—) Sol. A, (**—**) Sol. B, (**—)** Sol. C, (**—**) Sol. D.

To investigate the dissolution behavior of the metals in different bromide-based electrolytes, potentiodynamic polarization measurements were performed at room temperature. Figure 1 illustrates the polarization curves obtained after 1 h immersion for the anodic and cathodic scans.

The strong acidification of KBr solution, from pH = 6 to pH = 0.3, and the supplementary addition of Br_2_ affect the electrochemical behavior of the investigated metals in different ways, as discussed below.

Figure 1 shows that, in most cases, the cathodic polarization curves exhibit limiting diffusion plateaus, while the anodic dissolution branches present either a Tafel behavior or surface passivation. Consequently, the evaluation of the corrosion kinetic parameters (i.e., corrosion current density, corrosion potential, anodic and cathodic Tafel slopes) by Tafel extrapolation method over a wide potential range would lead to uncertainties and error sources.

Instead, in the close vicinity of the open-circuit corrosion potential, it is expected that the Stern–Geary theory [19] could be applied. It considers that both the anodic and cathodic processes follow the Tafel law and the global current density *i* could be expressed by the following Equation:

*i* = *i_a_* + *i_c_* = *i*_corr_{e*^b^*^a (*E*−*Ecorr)*^ – e*^b^*^c (*E*−*Ecorr*)^} (1)

where *i_corr_* is the corrosion current density, *E_corr_* is the corrosion potential and *b_a_* and *b_c_* are the anodic and cathodic activation coefficients (V^−1^), respectively.

To yield quantitative approach, the electrochemical kinetic parameters (*i_corr_*, *E_corr_*, *b_a_* and *b_c_*) were then evaluated from the experimental data by a non-linear regression calculation near zero overall current. A user-defined function of “Non-linear least squares curve fitting” of Origin 9.3 software (OriginLab Corporation, Northampton, MA, USA) based on Equation (1) was applied in the potential domain limited to ± 50 mV with respect to *E_corr_* [20]. In all cases, the correlation factor R^2^ varied within 0.9828–0.9998, indicating good fitting results.

**Figure 2:**
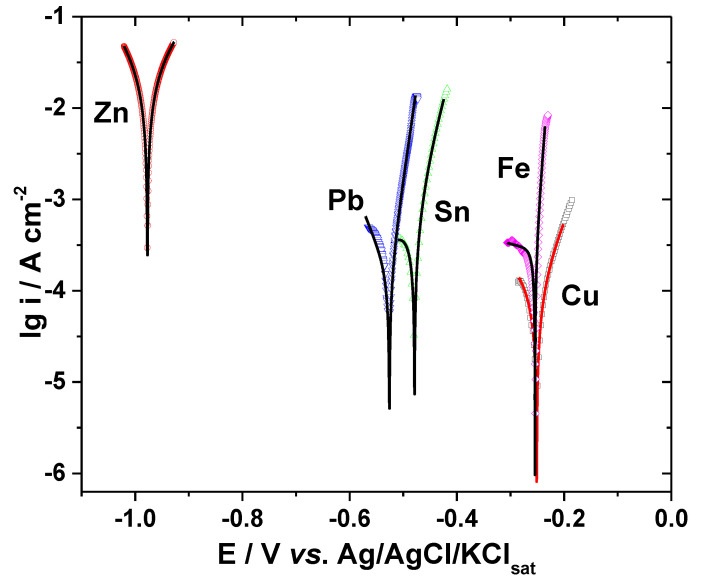
Polarization curves of various metals in Sol. C. Symbols, experimental data; lines, calculated data by Stern–Geary.

As an example, Figure 2 presents the polarization curves obtained by regression calculation in Sol. C overlaid on the experimental data represented by symbols.

The activation coefficients were linked to Tafel slopes, β, by the following Equation:

(2) $$b=\frac{2.303}{\beta }$$

The calculated corrosion kinetic parameters corresponding to metal corrosion in different bromide-based electrolytes are illustrated in Table 1.

In the case of Cu, the electrochemical behavior was found to be rather similar in all investigated electrolytes, as shown in Figure 1a. A very slight increase of the anodic and cathodic current densities could be noticed at high potentials in strong acidic KBr solution.

The addition of increasing Br_2_ concentrations shifts the *E_corr_* towards more positive values and markedly increases the corrosion current density (Table 1). For instance, the *i_corr_* value calculated in Sol. C is almost 7.5 times higher than that obtained in Sol. B. This corelates with an increased corrosion rate of Cu in the solution containing 0.01 M Br_2_. However, as shown in Figure 1a, the addition of Br_2_ mainly accelerates the cathodic reduction of the dissolved oxygen, which is under diffusion control, while the anodic dissolution of Cu is less affected. The increasing of the cathodic current density observed in the presence of bromide could be due to a reduction of the molecular Br_2_ that takes place in parallel with the oxygen reduction [21].

The anodic behavior of Cu in bromide-based electrolytes agrees with the results previously reported by other authors [22,23,24]. Thus, an apparent Tafel region with a slope of around 70 mV dec^−1^ could be observed on the anodic polarization curves obtained in the absence and presence of Br_2_. The anodic process occurring in this region might be related to the fast transformation of Cu into Cu^+^ ions, followed by the formation of soluble CuBr_2_^−^ complexes [22,23]. As the potential becomes more positive, an anodic current peak appears at about −60 mV vs. Ref on the polarization curves obtained in brominated solutions. The subsequent decrease of the current density noticed in Figure 1a might be associated to the formation of a porous and insoluble layer of CuBr on the electrode surface [24]. Finally, a region of sudden increase in the current density appears to be due to the presence of the aggressive Br^−^ ions reducing the adhesion properties of the adsorbed CuBr layer and leading to formation of soluble CuBr_2_^−^ complexes, which might suffer dissolution into the bulk solution. It has also been reported that, at high potential values (>0.2 V vs. Ref), the presence of CuO on the metallic surface is rather common, among the predominant Cu^2+^ compounds [24].

As expected, Zn presents the most negative potential (−0.97 V vs. Ref) and the highest anodic activity in all investigated electrolytes (Figure 1b). In near neutral KBr solution, a well-defined cathodic diffusion plateau was observed on the polarization curves due to the oxygen reduction reaction, while the anodic current density increases exponentially for about four orders of magnitude, within a potential range of +50 mV from *E_corr_*, with an apparent anodic Tafel slope of 12.6 mV dec^−1^, slightly lower as compared to a previously reported value [25].

A marked shift in both cathodic and anodic branches of the polarization curves towards higher current densities could be noticed in the strong acidic electrolytes (Figure 1b), suggesting an important enhancement of Zn corrosion. Although the calculated *E_corr_* values in different electrolytes are rather similar (Table 1), the corrosion current density significantly increases as the solution’s pH decrease.

The changes in the anodic Tafel slopes suggest that the dissolution mechanism of Zn might occur by a different pathway in strong acidic KBr solution as compared to neutral one. Nevertheless, the addition of Br_2_ slightly accelerates the electrochemical reactions, but does not modify the mechanism of Zn corrosion. The large estimated cathodic Tafel slopes are consistent with the hypothesis of a diffusion limited reduction reaction.

In Sol. A, the region of active Sn dissolution observed at low potentials is followed by a narrow semi-passive region [26], characterized by lower anodic current density values (Figure 1c), due to the presence of a thin film of tin oxide on the electrode surface [27]. The passivity breakdown observed at −0.32 V vs. Ref could be related to the pitting induced by Br^−^ ions at the oxide–electrolyte interface [28].

As revealed in Figure 1c, the Sn dissolution process is significantly favored by the electrolyte acidification. Moreover, the cathodic oxygen reduction reaction appears to be greatly enhanced by Br_2_ addition. In Table 1, an increase of *i_corr_* values can be noticed in all investigated acidic solutions, while the *E_corr_* values are shifted towards less noble values. The highest negative shift of *E_corr_* was observed in the presence of 0.01 M Br_2_. At this concentration, the corrosion current density presents its greatest value, which is about 29 and 285 times higher than *i_corr_* values obtained in Sol. B and Sol. A, respectively. The acceleration of Sn dissolution in strong acidic electrolytes might be due to adsorption of aggressive Br^−^ ions at active sites of Sn surface, followed by the formation of soluble complexes transferred into the solution. As previously stated by Johnson and Liu [29], such complexing processes decrease the metallic ions concentration retarding the formation of the oxide passive layer. However, at around −0.35 V vs. Ref, a minimum peak current could be observed on the polarization curves obtained in strong acidic KBr solution, both in the absence and in the presence of Br_2_. Its interpretation is rather uncertain; some authors attributed it to the formation of Sn(IV) species [26], while others to some complex compounds formed on the metallic surface [28]. Nevertheless, the increased aggressiveness of Sol. C induces pitting corrosion, which explains the sharp increase of the current density values at high potentials (Figure 1c).

The anodic Tafel slopes present similar values regardless of the electrolyte’s nature, suggesting that the Sn dissolution mechanism is rather similar.

As shown in Figure 1d, the polarization curves of Pb were shifted towards more positive values in strong acidic KBr solutions, and even more in the presence of Br_2_. Although not very clearly seen in Figure 1d, a small transition region appears at around −0.58 V vs. Ref on the polarization curves obtained in Sol. A; it may correspond to the formation of a passivating layer on the metallic surface, which further dissolves as the potential become more positive. It may be inferred that the reactivation region is associated with the formation of soluble lead species, subsequent to the passivating step [30].

In strong acidic solutions, regardless the addition of bromine, the superficial corrosion products layer cannot impart passivity because its solubility is relatively high [30] and Pb surface dissolves readily leading to Pb^2+^ formation [31]. A similar behavior was previously noticed for Pb corrosion in chloride electrolytes [30].

At about −0.47 V vs. Ref, a sharp increase of the current density could be observed on the anodic part of the polarization curves in all investigated electrolytes. This decrease of the current density may be due to the formation of a layer corrosion products on Pb surface.

In Table 1, it is clear that the overall corrosion process of Pb is enhanced by the aggressiveness of bromine. Thus, the calculated *i_corr_* value reached 144.4 μA cm^−2^ in the presence of 0.01 M Br_2_, which is almost four time greater than the value obtained in Br_2_-free acidic solution (37.8 μA cm^−2^).

As previously reported, Pb corrosion in aqueous solutions might be represented by a two-electron transfer reaction [31]. Depending on the solution’s nature, this reaction may occur in a sequence of steps, in which the formation of monovalent intermediate is possible [31], as suggested by the low values of *β_a_* calculated in strong acidic solutions, in the absence and in the presence of Br_2_ (Table 1).

In the case of iron, Br^−^ ion might adsorb on the electrode surface promoting the metal dissolution [32]. Consequently, no passivation of Fe surface was observed in the studied experimental conditions (Figure 1e). Particularly, the Br_2_ addition significantly enhances both the active dissolution of Fe and cathodic oxygen reduction, which is under diffusion control. Thus, the corrosion current density in the presence of 0.01 M Br_2_ is more than 38 and 76 times higher as compared to *i_corr_* values calculated in Sol. B and Sol. A, respectively. Moreover, a displacement of *E_corr_* towards more noble values takes place in Br_2_-containing solutions (Table 1). The low values of the anodic Tafel slopes calculated in all acidic electrolytes advises that some adsorbed intermediates might be involved in Fe corrosion mechanism.

### 3.2. Electrochemical Impedance Spectroscopy

**Figure 3:**
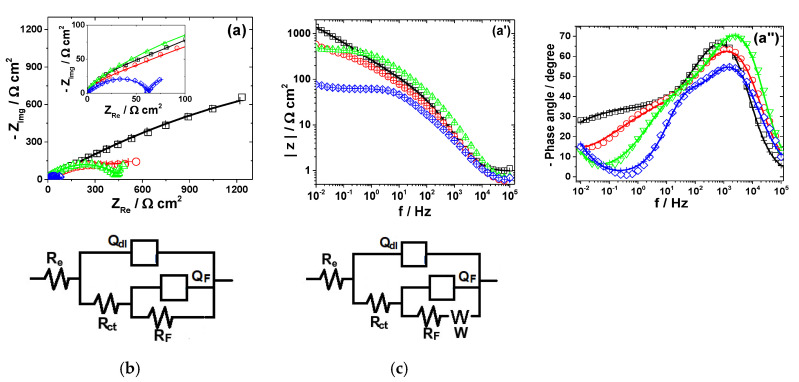
EIS results of Cu after immersion in different bromide-based electrolytes: Nyquist diagrams (**a**); Bode plot of |Z| vs. frequency (**a’**); and Bode plot of phase angle vs. frequency (**a”**). The equivalent electrical circuits used to fit the EIS data (**b**,**c**). Symbols represent the experimental data and lines with cross (—+—) correspond to the simulated spectra. Electrolytes: (□) Sol. A; (O) Sol. B; (◊) Sol. C; and (Δ) Sol. D.

**Figure 4:**
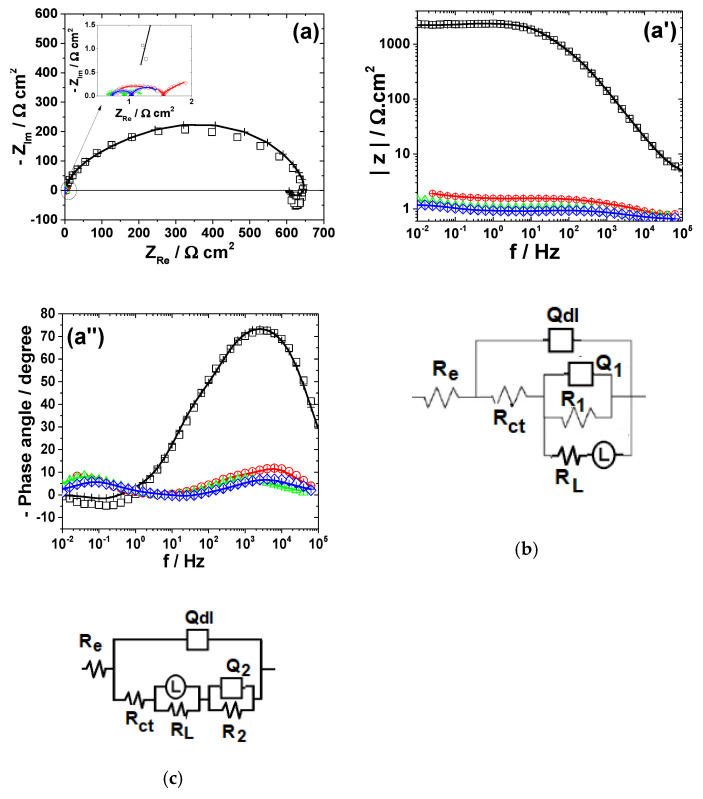
EIS results of Zn after immersion in different bromide-based electrolytes: Nyquist diagrams (**a**); Bode plot of |Z| vs. frequency (**a’**); and Bode plot of phase angle vs. frequency (**a”**). The equivalent electrical circuits used for EIS fitting (**b**,**c**). Symbols represent the experimental data and lines with cross (—+—) correspond to the simulated spectra. Electrolytes: (□) Sol. A; (O) Sol. B; (◊) Sol. C; and (Δ) Sol. D.

**Figure 5:**
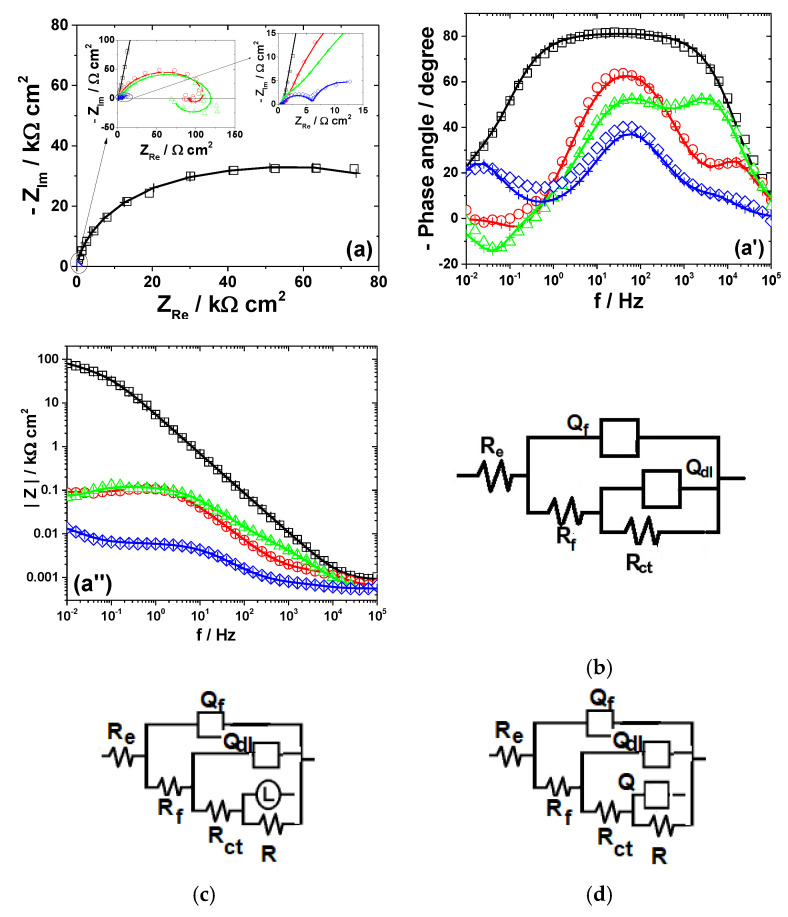
EIS results of Sn after immersion in different bromide-based electrolytes: Nyquist diagrams (**a**); Bode plot of |Z| vs. frequency (**a’**); and Bode plot of phase angle vs. frequency (**a”**). The equivalent electrical circuits used for EIS fitting (**b**–**d**). Symbols represent the experimental data and lines with cross (—+—) correspond to the simulated spectra. Electrolytes: (□) Sol. A; (O) Sol. B; (◊) Sol. C; and (Δ) Sol. D.

**Figure 6:**
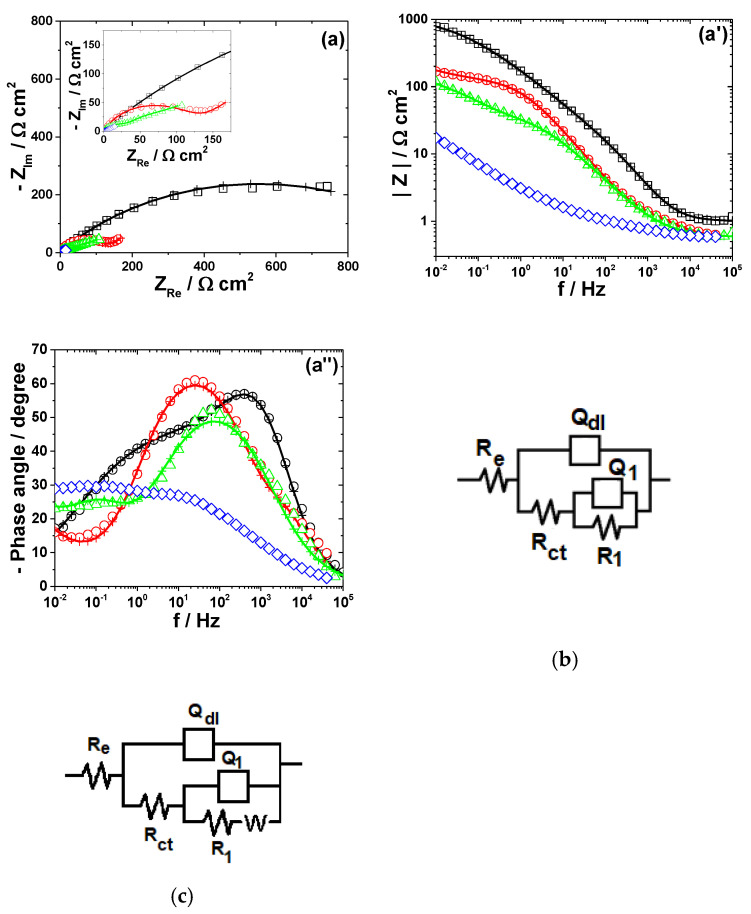
EIS results of Pb after immersion in different bromide-based electrolytes: Nyquist diagrams (**a**); Bode plot of |Z| vs. frequency (**a’**); and Bode plot of phase angle vs. frequency (**a”**). The equivalent electrical circuits used for EIS fitting (**b**,**c**). Symbols represent the experimental data and lines with cross (—+—) correspond to the simulated spectra. Electrolytes: (□) Sol. A; (O) Sol. B; (◊) Sol. C; and (Δ) Sol. D.

**Figure 7:**
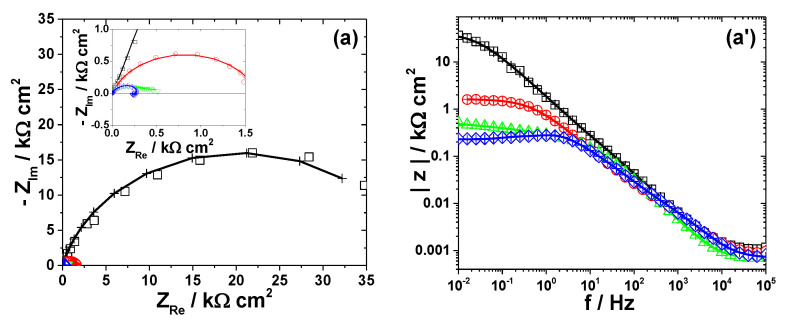

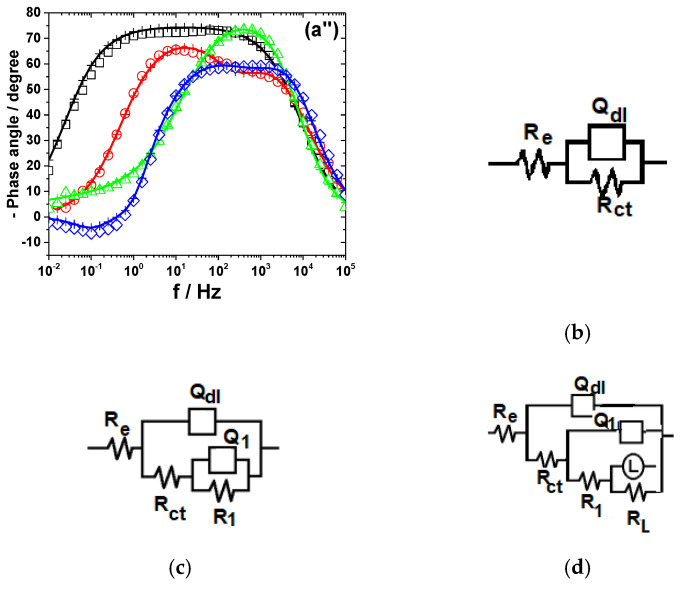
Displays the impedance diagrams of Fe immersed in the studied electrolytes. EIS results of Fe after immersion in different bromide-based electrolytes: Nyquist diagrams (**a**); Bode plot of |Z| vs. frequency (**a’**); and Bode plot of phase angle vs. frequency (**a”**). The equivalent electrical circuits used for EIS fitting (**b**–**d**). Symbols represent the experimental data and lines with cross (—+—) correspond to the simulated spectra. Electrolytes: (□) Sol. A; (O) Sol. B; (◊) Sol. C; and (Δ) Sol. D.

The electrochemical dissolution behavior of the metals in different bromide-based systems was further investigated by electrochemical impedance spectroscopy (EIS). The impedance measurements were conducted at the open-circuit potentials and the obtained results are displayed as Nyquist diagrams and Bode plots in Figure 3, Figure 4, Figure 5, Figure 6 and Figure 7. The insets in Figure 3, Figure 4, Figure 5, Figure 6 and Figure 7 present some of the Nyquist diagrams in enlarged scale.

As shown in Figure 3, Figure 4, Figure 5, Figure 6 and Figure 7, disregarding the metal, an important decrease of the impedance magnitude could be observed in strong acidic KBr solution as compared to the near neutral one, and even more in the presence of Br_2_, which is consistent with an increased dissolution rate of all metals in brominated electrolytes, in agreement with the potentiodynamic polarization results.

Before attempting to fit the experimental impedance data with electrical equivalent circuits, an evaluation of the EIS data validity with respect to linearity, causality and stability was carried out. For this purpose, Kramers–Kronig relations were applied to several experimental impedance data by transforming the real axis into the imaginary axis and the imaginary axis into the real axis and then comparing the transformed quantities with the corresponding experimental data [24]. The satisfactory agreement between the measured and transformed values (within the range of experimental errors) demonstrates that the experimental impedance data are consistent with K-K transformations and therefore stable, as shown in Section S1 (Figure S1).

EIS results obtained in Sol. A, Sol. B and Sol. C were further analyzed by numerical simulation, using different equivalent electrical circuits that allow estimation of the *R*-*Q* parameters corresponding to the electrochemical systems under investigation and might validate the proposed reaction mechanisms [31].

The capacitive contributions of the impedance plots were simulated using constant phase elements (CPE), represented by the terms *Q* and *n*, instead of a pure capacitors (*C*) due to the non-ideal behavior of the metallic surfaces. The semicircle depression is often explained by the surface heterogeneity, caused by surface roughness, formation of porous layers, defects in the crystal lattice, variations in the properties or compositions of the surface layers, adsorption and presence of impurities [33].

The impedance of CPE is given by [34]:

*Q* = *Z*_CPE(*ω*)_ = [*C*(*jω*)*^n^*]^−1^ (3)

where *Q* represents a pre-exponential factor, which is a frequency-independent parameter with dimensions of Ω^−1^ cm^−2^ s*^n^*; *j* is an imaginary number; *ω* = *2πf* is angular frequency in rad·s^−1^; and *n* is the exponent which defines the character of frequency-dependence (−1 ≤ *n* ≤ 1).

The values of the pseudo-capacitances (C) associated with CPEs were recalculated using the Equation:

(4) $$C={\left(R^{1-n}Q\right)}^{1/n}$$

The equivalent electrical circuits used for experimental EIS data simulation are illustrated together with the impedance diagrams in Figure 3, Figure 4, Figure 5, Figure 6 and Figure 7.

The used equivalent electrical circuits reproduce properly all experimental impedance, as could be seen in Figure 3, Figure 4, Figure 5, Figure 6 and Figure 7, where good overlapping between the measured data (represented by scattered symbols) and calculated ones (denoted by —+—) were obtained for all studied systems. The quality of the fitting procedures was also evaluated by the Chi-squared (χ^2^) values situated in 10^−4^–10^−3^ range and by the error percentages corresponding to each parameter of the used electrical circuits, which were mostly below 10%.

Since the physical meaning of the time constants used for impedance simulations was different, depending on the studied metal and electrolyte characteristics, a detailed discussion on their interpretation will be further provided.

#### 3.2.1. Copper

The experimental EIS spectra obtained from Cu surface exposed to the bromide-based electrolytes are presented in Figure 3.

As shown in Figure 3a, the impedance diagrams of Cu in the investigated electrolytes are characterized by a semicircular appearance with badly separately capacitive loops. Although not clearly seen in Figure 3a, two-time constants are necessary to suitably reproduce the experimental data obtained in Sol. A and Sol. B. The equivalent electrical circuit [35,36] depicted in Figure 3b was adopted for these data simulations. In the equivalent circuit in Figure 3b, *R_e_* represents the electrolyte resistance, the high-frequency parameters (*R_ct_*−*Q_dl_*) correspond to the charge transfer resistance and double layer capacitance and the low frequency circuit represented by *R_F_*−*Q_F_* was ascribed to a redox process involving the corrosion products (i.e., Cu_2_O and CuO) accumulated at the interface, based on the following reactions [20]:

Cu ↔ Cu^+^_ad_ + e^−^

Cu^+^_ad_ → Cu^2+^ + e^−^

The impedance spectra obtained in the presence of Br_2_ were fitted to the equivalent circuit in Figure 3b. It contains an additional Warburg impedance to account for the appearance of the diffusion tail observed at lowest frequency region in the EIS diagrams collected from Br_2_-containing electrolytes.

The *R*-*Q* parameters for Cu dissolution in different bromide-based electrolytes obtained by fitting the equivalent electrical circuits are given in Table 2.

As shown in Table 2, the charge transfer resistance, *R_ct_*, values significantly decrease as the electrolyte’s pH becomes acidic and even more in the presence of 0.01 mM Br_2_. This decay of *R_ct_* is accompanied by an increase of the double layer capacitance *C_dl_*. The corrosion process is faster in strong acidic electrolytes and Cu surface becomes coarser, leading to higher *C_dl_* values. At the same time, the faradaic resistance calculated in Sol. C is more than 9 and 75 times lower than the *R_F_* values obtained in Sol. B and Sol. A, respectively. These results advise for an increased electrochemical reactivity of Cu in brominated electrolyte, in agreement with the polarization measurements.

#### 3.2.2. Zinc

The experimental EIS spectra obtained from Zn electrode exposed to the bromide-based electrolytes are presented in Figure 4.

As shown in Figure 4, the impedance plots obtained during Zn dissolution in near neutral and strong acidic KBr electrolytes present different features, which indicates that some changes in the reaction mechanisms with the solution pH might occur.

Although the corrosion behavior of Zn in bromide [37] or bromine-containing solutions is scarcely reported [38], its dissolution mechanism in chloride-based electrolytes was subject to numerous investigations. The previously reported reaction models [39,40,41,42] involve several parallel steps of dissolution and three adsorbed species (Zn_ad_^+^, ZnOH_ad_ and Zn_ad_^2+^), depending on Zn surface preparation and the experimental conditions (pH, oxygen concentration and the presence of different oxidized species, i.e., oxide or hydroxide, on Zn surface) [43]. For example, Cachet and Wiart [39] proposed a reaction scheme for the dissolution of Zn in aerated ZnCl_2_ and NH_4_Cl which involves two parallel steps, stimulated by the presence of chloride ions:


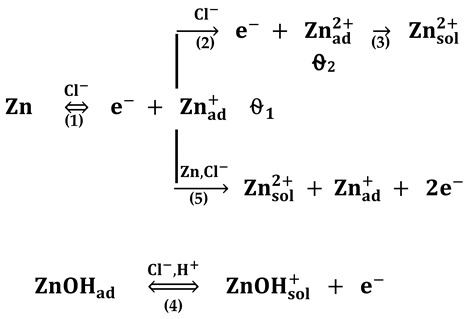


The major path (2) is catalyzed by Zn^+^_ads_. The minor path (1), is much more dependent on the diffusion of the chloro-zinc species than the major one. The formation of ZnOH_ads_ is considered a side reaction, which is caused by the chemical oxidation of Zn by the electrolyte. Close to the rest potential, additional difficulties come from the competition between the slow chemical formation of the oxidized protective layer and its elimination under the effect of Zn dissolution [41].

**Figure 8:**
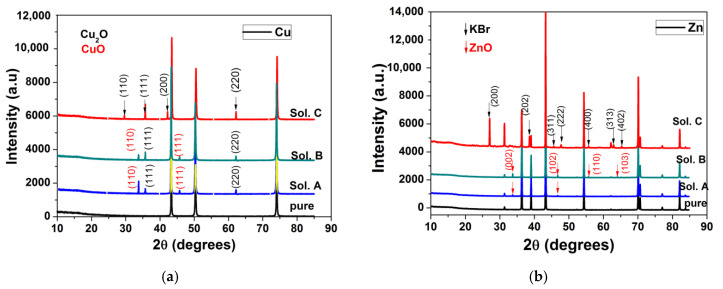

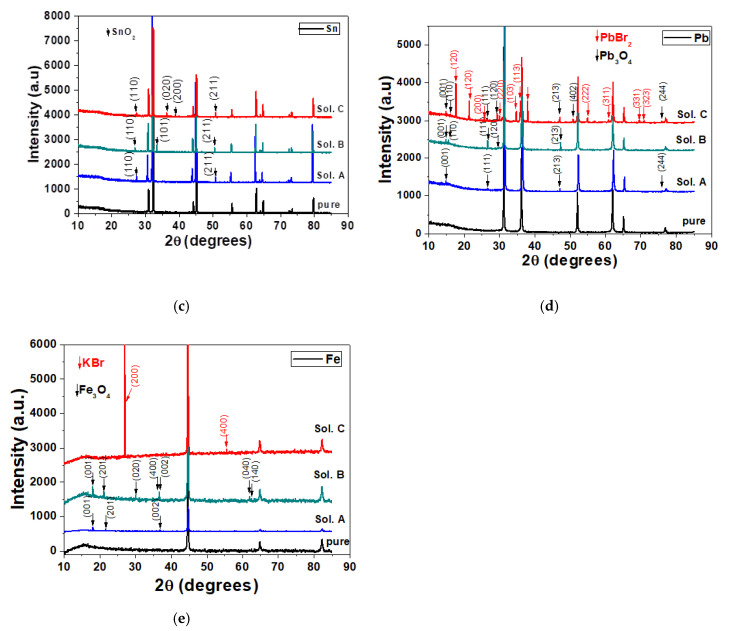
XRD diffractograms collected before and after 120 h immersion of electrodes in different bromide-based electrolytes: Cu (**a**); Zn (**b**); Sn (**c**); Pb (**d**); and Fe (**e**).

This study found that the impedance corresponding to Zn corrosion in Sol. A could be suitably represented using two time constants under capacitive relaxation and an inductive contribution at low frequency, according to the electrical circuit in Figure 4b. The two capacitive loops were ascribed to the charge transfer process (*R_ct_*−*Q_dl_*) and the coverage relaxation of an adsorbed Zn_ad_^+^ intermediate (*R*_1_−*Q*_1_), respectively. The appearance of an inductive loop (*R_L_*−*L*) in the low frequency-end was assumed to be due to the relaxation of an oxidized intermediate species (i.e., ZnOH_ad_); its formation might express the chemical oxidation of Zn by the electrolyte [41]. This very thin layer of oxidized Zn species might present some protective properties on the metallic surface [41,42,44]. As discussed below, the low value of *C_dl_* (5.5 μF cm^−2^) obtained in Sol. A is consistent with the presence of a protective layer on Zn surface. Furthermore, the presence of a thin layer of ZnO on the metallic surface was identified in the XRD pattern of Zn exposed for 120 h to Sol. A, according to the data in Figure 8b.

The decrease in the solution pH significantly favors the Zn dissolution process, hindering the formation of the oxidized species on the metallic surface. This might explain the vanishing of the inductive loop at low frequency [25]. Nevertheless, the appearance of a small inductive loop at intermediate frequency (~10 Hz) between two capacitive contributions could be noticed in strong acidic electrolytes, both in the absence and in the presence of Br_2_ (Figure 4a) [25,41]. The similar features of the impedance diagrams obtained in strong acidic electrolytes, regardless of the presence of Br_2_, confirm that a similar dissolution mechanism of Zn is involved, in agreement with the polarization measurements. Thus, the inductive loop was attributed to the relaxation of the adsorbed Zn_ad_^+^ intermediate, while the low-frequency capacitive semicircle might be associated to the formation of Zn_ad_^2+^ species during the dissolution reaction [45,46]. However, as shown in Figure 4a, the maximum frequency of the second capacitive loop is very low, which allowed us to assume that the formation and consumption of the adsorbed intermediate occurs in parallel with the main dissolution path [46].

The impedance diagrams of Zn dissolution in strong acidic KBr solutions were accurately simulated using the equivalent circuit in Figure 4c and several calculated *R*-*Q* parameters are presented in Table 3.

As shown in Table 3, the charge transfer resistance *R_ct_* presents a similar pH dependence as the *i_corr_* values calculated from polarization measurements; it decreases from about 322.9 Ω cm^2^ in Sol. A to 0.87 Ω cm^2^ in Sol. B, confirming that Zn suffers severe corrosion in strong acidic KBr electrolyte. The addition of Br_2_ accelerates to some extent the dissolution process, as proved by the *R_ct_* value which is more than three orders of magnitude lower in the presence of 0.01 M Br_2_, as compared to its absence. Additionally, the significant increase of *C_dl_* values in Br_2_-containing electrolytes corroborates the formation of coarse surface during Zn dissolution, as revealed by SEM micrograph (results shown in Figure S2).

#### 3.2.3. Tin

The impedance spectra of Sn exposed to different bromide/bromine electrolytes are illustrated in Figure 5.

As shown in Figure 5a, the Nyquist diagram obtained for Sn corrosion in near neutral KBr solution consists of a wide capacitive contribution, with a large imaginary part at the lowest frequency. The attempts to represent the EIS data of Sn exposed to Sol. A by one time constant circuit were found inappropriate and two time constants were required to accurately reproduce the experimental results. As revealed in Figure 5b, the model adopted for simulation includes one couple, *R_f_*−*Q_f_*, ascribed to the capacitance and resistance of a thin passive layer composed of tin oxides, formed on the metallic surface [47] and the *R_ct_*−*Q_dl_* parameters corresponding to the charge transfer resistance and double layer capacitance. The XRD measurements performed after 5 h immersion of Sn in Sol. A did not detected the presence of any corrosion products (results not shown here) because their amount was too small [48]. Instead, the occurrence of SnO_2_ on the surface was evidenced in the XRD diffractogram obtained at longer exposure time (Figure 8c).

An additional inductive loop at low frequencies is observed in EIS spectra corresponding to Sn corrosion in strong acidic KBr electrolytes, both in the absence and in the presence of 0.001 M Br_2_ (Figure 5c). Several models of equivalent circuits were attempted to fit these experimental data. The best agreement between experiment and fitting was obtained using the equivalent circuit in Figure 5c. It contains an additional (*R_L_*−*L*) couple proposed to account for the low-frequency inductive time constant, which might be attributed to the pitting corrosion induced by Br^−^ ions on Sn surface [49]. However, as the concentration of Br_2_ increases to 0.01 M, the low-frequency inductive loop becomes capacitive and the equivalent circuit in Figure 5d was used to simulate the corresponding impedance.

Table 4 illustrates the equivalent circuit parameters calculated for Sn dissolution in the investigated electrolytes.

The strong passivation of tin in Sol. A is confirmed by the high values of *R_f_* and *R_ct_* (Table 4). As the electrolyte become more acidic, the resistance of the thin SnO_2_ layer formed on Sn surface *R_f_* and the charge transfer resistance of the underlying metal *R_ct_* significantly decrease. In parallel, important increases of *C_f_* and *C_dl_* values were observed in Sol. C. These results correlate with a passivity breakdown in strong acidic electrolytes [50], leading to higher corrosion rates and much rougher surfaces with enlarged areas. The presence of 0.01 M Br_2_ additionally accelerates the active Sn dissolution, in accordance to the outlined conclusions of the potentiodynamic measurements.

#### 3.2.4. Lead

Figure 6 presents the impedance spectra measured on Pb electrode exposed to different bromide-based electrolytes and the electrical equivalent circuits used for data simulation.

The development of more than one time constant could be noticed in all impedance spectra corresponding to Pb corrosion (Figure 6). The Bode plot for Pb dissolution in Sol. A shows two maxima in the phase, which were interpreted according to the equivalent circuit in Figure 6b. The first time constant at high frequencies was ascribed to the double layer capacitance *Q_dl_* connected in parallel with the charge transfer resistance *R_ct_*, while the second one corresponds to some adsorbed species that contributes to the formation of the corrosion layer [51]. An additional Warburg tail associated to a diffusion process at the metallic surface was noticed in the impedance diagram of Pb exposed to Sol. B. Accordingly, the corresponding EIS data were interpreted in terms of the equivalent circuit depicted in Figure 6b and the calculated R-Q parameters are shown in Table 5.

As expected, the fast dissolution of Pb in strong acidic brominated electrolytes was confirmed by the low values of *R_ct_* and *R*_1_ (Table 5). The attempts to simulate the EIS data obtained for Pb corrosion in solutions containing Br_2_ with different electrical equivalent circuits did not allow a satisfactory agreement between the used models and experimental results. A possible explanation could be related to the complexity of the electrochemical processes taking place at Pb surface in the presence of Br_2_, leading to the formation of various compounds, i.e., Pb_3_O_4_ and PbBr_2_, which complicate the corrosion mechanism. However, the decrease of the overall impedance and phase angle in Sol. C noticed in Figure 6 correlates with an important increase of Pb corrosion rate in brominated electrolytes.

#### 3.2.5. Iron

The Nyquist diagram of Fe exposed to Sol. A shows a wide depressed capacitive contribution, which was interpreted according to the Randles circuit model in Figure 7b [52]. Instead, two time constants are clearly visible in the Bode plots obtained in Sol. B and Sol. D, suggesting that the mechanism of Fe corrosion in strong acidic electrolytes might involve one adsorbed intermediate (i.e., Fe_ad_^+^) covering the electrode surface [53]. The relaxation process of the adsorbed intermediate was represented by *R*_1_−*Q*_1_ circuit nested in the typical *R_ct_*−*Q_dl_* network, as revealed in Figure 7c.

An inductive loop in the low-frequency domain could be observed for Fe corrosion in Sol. C. It could originate from the relaxation process of some adsorbed intermediate species formed on Fe surface [54]. The equivalent circuit in Figure 7d, containing the *R_L_*−*L* inductive elements, was used for these data simulations. It should be noted that similar impedance results were obtained in the case of Fe dissolution in NaCl solutions of pH = 0, 1, 2, 3, 4 [55].

Table 6 presents the values of R-Q parameters obtained by fitting the experimental data corresponding to Fe corrosion in different bromide-based electrolytes to the equivalent electrical circuits in Figure 7.

As expected, the electrochemical dissolution of Fe is enhanced by the decrease of solution pH, as revealed by the low values of *R_ct_* and *R*_1_ obtained in Sol. B (Table 6). The addition of Br_2_ further accelerates the corrosion process, i.e., *R_ct_* and *R*_1_ values are more than six times lower in Sol. C as compared to Sol. B.

### 3.3. Ex-Situ Examinations

#### 3.3.1. XRD Measurements

The XRD spectra of Cu, Zn, Sn, Pb and Fe obtained after 120-h immersion in different bromide-based electrolytes are shown in Figure 8.

Prior to XRD measurements, the above-mentioned metallic samples were mechanically and chemically cleaned to remove any traces of corrosion from their surface. XRD diffractograms for all cleaned samples (pure curves in Figure 8) show that they are pure, without traces of oxides or other compounds on their surface.

The long immersion of the metals in different bromide-based electrolytes induces structural modifications for all electrodes, as shown in their XRD diffractograms. In the case of Cu electrode immersed in Sol. A and Sol. B, the XRD diffractograms shows a combination of CuO and Cu_2_O phases. By exposing the Cu electrode to Sol. C, the corrosion products change their composition and XRD diffractograms show only peaks related to the Cu_2_O phase. In the case of Sn, the additional diffraction peaks appearing in the diffraction pattern after 120 h were assigned to SnO_2_ and their intensity increases in Sol. C. In the case of Pb the influence of the Sol. C is more obvious. The peaks in the XRD diffractograms were indexed with red arrows (for PbBr_2_ phase) and black arrows (for Pb_3_O_4_ phase). The predominance of PbBr_2_ becomes visible.

The XRD diffractograms of Zn and Fe after immersion in Sol. A and Sol. B reveal the presence of corrosion products, i.e., Fe_3_O_4_ and ZnO, respectively. Instead, the XRD diffractograms for Fe immersion in Sol. C show only the peaks corresponding to KBr.

From the XRD diffractograms obtained on the metal electrodes, a preferential orientation tendency of the crystallites of the corrosion products was observed according to certain crystallization planes. To study the effect of dissolution of metals in different bromide-based systems on the preferential orientation of crystallites, we assessed the X-ray diffractograms obtained on samples exposed to these solutions for 120 h. The relative values of the intensities of the diffraction peaks for all samples are presented in Table 7.

In the case of Cu and Sn electrodes, a preferential orientation of the crystallites of the corrosion products is observed according to the diffraction planes (111) for Cu_2_O and (110) for SnO_2_. The increase in the intensity ratios after these diffraction planes indicates that the corrosion products become more stable [56], in agreement with potentiodynamic polarization and EIS measurements. In the case of Pb electrodes, the small variation of the I_110_/I_211_ ratio of the intensities around 1 for Pb_3_O_4_ is probably due to the fact that the formation of the corrosion product PbBr_2_ is more accentuated, as can be seen in Figure 8c.

For the Fe and Zn electrodes, a preferential orientation on the c direction of the crystallites of the corrosion products is observed, according to the diffraction planes (001) for Fe_3_O_4_ and (002) for ZnO. The decrease in the solution pH significantly influences the Fe and Zn dissolution process, preventing the formation of oxidized species on the metallic surface, consistent with EIS measurements.

#### 3.3.2. XPS Measurements

To get deeper insight into the corrosion processes taking place on the metallic surfaces after 60–120 min immersion in Sol. C, X-ray photoelectron spectroscopy was used. XPS might act as a valuable tool for studying the initial stages of corrosion, when the layer of formed corrosion products may be too thin for bulk sensitive methods such as for X-ray diffraction.

##### Cu Samples

**Figure 9:**
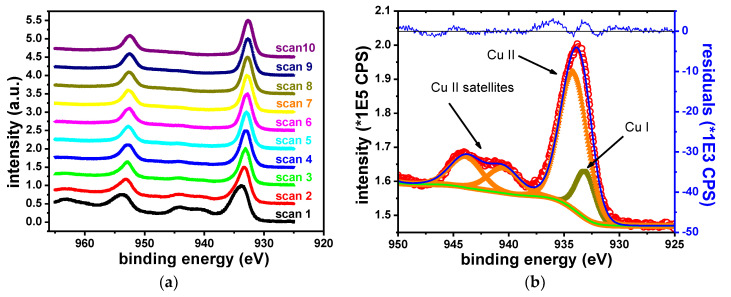
Copper sample degradation (**a**) and fit of the initial narrow scan (**b**).

The elements identified in the survey scans of Cu samples are Cu, O, Br, K, C and Si. As expected, sample degradation due to X-ray exposure was observed (Figure 9). The sample degradation was measured by repeating the Cu 2p narrow scan acquisition for 10 iterations and subsequently fitting the spectra. Figure 9 shows the changes in the Cu 2p narrow scan data due to sample degradation and the fit of the initial dataset (Iteration 0). The peak shift observed on the left side of the figure is ascribed to charging effects. At least two contributions are required to fit the data, which were ascribed to Cu^2+^ and Cu^1+^, respectively.

Table 8 gives the binding energies of the two contributions and their attributed oxidation states. The initial spectrum shows satellite features at the following binding energies: 940.5, 943.9 and 962.1 eV, which are specific to Cu^2+^ state [57]. The Cu 2p_3/2_ contribution associated with Cu^2+^ was observed at 934.2 eV. The Cu 2p_3/2_ contribution associated with Cu^1+^ was observed at 932.6 eV, in agreement with formerly reported results [58,59,60]. With the prolonged X-ray exposure, Cu^2+^ satellite features vanish. The intensity of the Cu 2p_3/2_–Cu^2+^ peak decreases. Conversely, the intensity of the Cu 2p_3/2_–Cu^1+^ peak increases. This indicates the reduction of the Cu^2+^ component. The degradation of Cu^2+^ compounds during XPS measurements is a well-known occurrence [44,61].

The ratio of the two oxides was estimated by adding the contribution of the Cu^2+^ satellite peaks to the contribution of the main Cu^2+^ peak. Table 9 shows the quantification results.

The O1s peak presents three components which can be ascribed to organic contaminations and to metallic oxides, respectively. The component ascribed to metallic oxides is observed at 530.1 eV, in accordance with the values previously reported for Cu_2_O, which are in the range of 530.2–530.7 eV [62,63].

**Figure 10:**
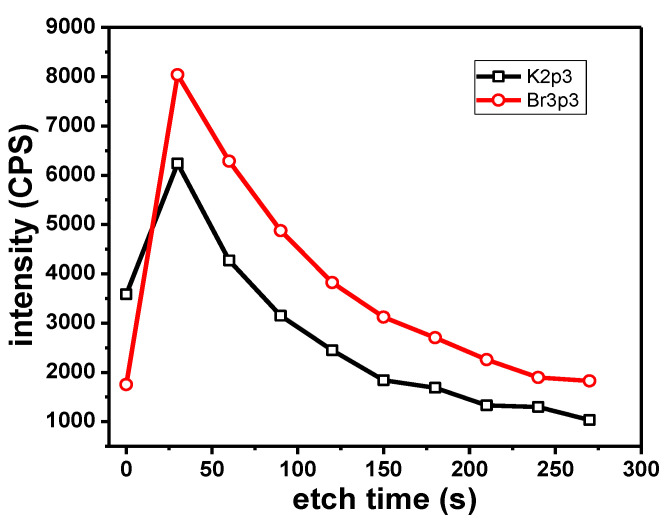
Variation of K (black) and Br (red) peak intensity with etching time.

The presence of K along with Br could indicate the presence of recrystallized KBr traces on the sample. The measured B.E. of the K2p_3/2_ and Br3p_3/2_ electrons are consistent with the expected values for KBr. The K2p_3/2_ peak is observed at 292.9–293.0 eV and the Br3p_3/2_ peak is observed at 182.1 eV. Typical values reported in the literature are in the range of 292.9–293.1 eV for K in KBr [64,65] and 182.1 eV for Br in NaBr [66]. Depth profile experiments (Figure 10) showed a parallel variation of peak intensity for the K2p_3/2_ and the Br3p_3/2_ photoelectrons with the etching time. This further indicates the presence of KBr.

##### Zn Samples

The main elements identified in the case of the Zn sample are Zn, K, Br, C and O. The C1s peak of the adventitious species was observed at 285.0 eV. Zn is observed in the Zn^2+^ oxidation state on the “as received” sample. The Zn 2p_3/2_ peak appears at 1022.5 eV, in agreement with previously reported values for ZnO or Zn(OH)_2_ [44,67]. During depth profile experiments, the peak shape of the Zn 2p and of the Zn LMM electrons indicates the emergence of metallic Zn. No metallic Zn is observed before sputtering.

**Figure 11:**
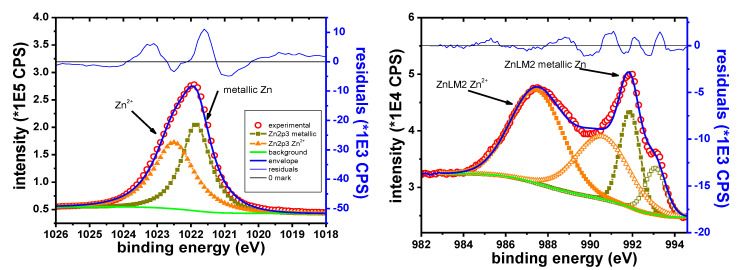
Zn 2p narrow scan (**left**), Zn LMM narrow scan (**right**).

Figure 11 shows the narrow scans for the two types of electrons. The XPS electrons are shown after 150 s of etching. The X-ray induced Auger electrons are presented after 120 s of etching. In both examples, oxide and metallic contributions are clearly visible. This highlights the presence of the 2+ oxide state before etching.

Table 10 shows the B.E. and the K.E. values corresponding to the two oxidation states.

The measured ratios of Zn:O could be useful in assigning the chemical composition. The Zn_oxide_: O atomic ratio is 49.7:50.3. This is close to the 1:1 expected ratio for ZnO.

**Figure 12:**
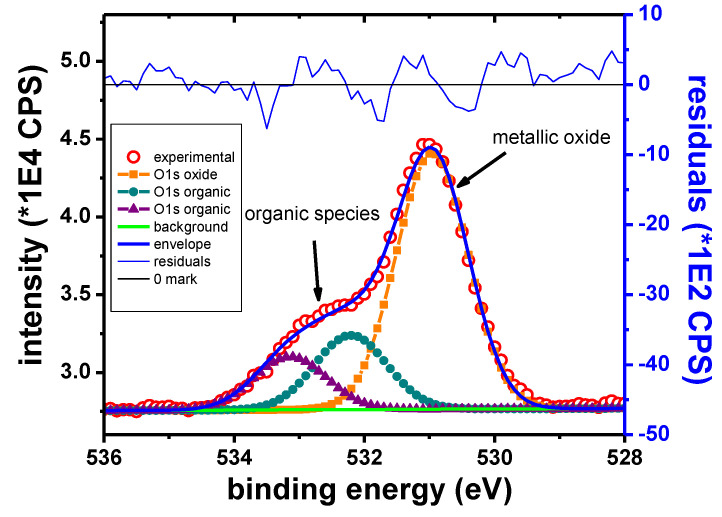
O1s narrow scan, Zn sample.

Figure 12 shows the O1s scan after 270 s of etching. The peak at 531.0 eV was attributed to Zn oxide, in accordance with other reported data [44,67]. The peaks from 532.2 and 533.1 eV correspond to adventitious organic species. The peak intensity in the case of the organic components is decreasing rapidly with sputtering, although the peaks remain detectable throughout the entire depth profile experiment.

K and Br are also observed for the Zn sample, but the two elements are removed from the sample at the same rate during the depth profile experiment. It could therefore be inferred that they belong to the same compound, namely KBr.

##### Sn Samples

**Figure 13:**
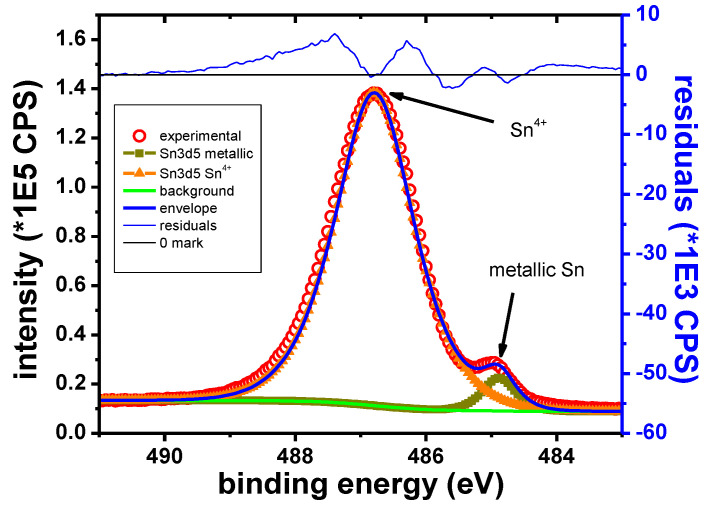
Sn 3d_5/2_ narrow scan, Sn sample.

In the case of the Sn sample, the main elements identified in the XPS survey spectra are Sn, O, K, Br and C. The Sn 3d_5/2_ narrow scan presented in Figure 13 shows two well separated peaks. These peaks are identified as corresponding to the metallic and Sn^4+^ states, respectively, according to their B.E values (Table 11).

**Figure 14:**
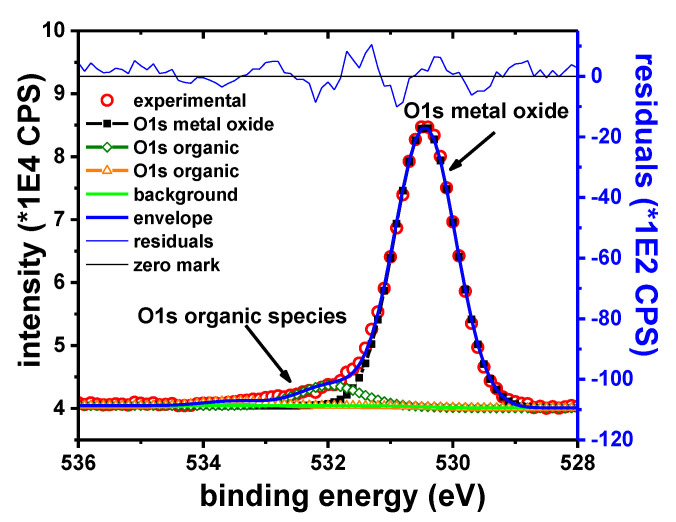
O1s narrow scan, Sn sample.

Figure 14 shows the O1s peak for the Sn sample after 15 s of etching. Contributions from organic compounds and the inorganic oxide are initially observed. The O1s peak attributed to metallic oxides was observed at 530.4 eV. During the depth profile, the organic contributions vanish rapidly. The inorganic contribution remains visible as long as the Sn^4+^ peak remains visible. This behavior is consistent with the presence of Sn oxide on the surface. Because Sn was found in Sn^4+^ state, it was assumed that the sample surface contains SnO_2_.

##### Pb Samples

The main elements observed in the XPS survey spectra of the Pb sample were only Pb, Br, C and O. The C1s peak is observed at 285.0 eV. C and O are ascribed only to the adventitious organic species. The intensity of these peaks is decreasing rapidly and at the same rate, suggesting that both elements belong to the same compounds.

**Figure 15:**
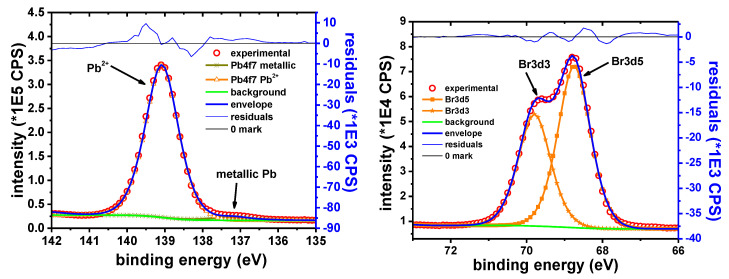
Pb 4f_7/2_ narrow scan (**left**); and Br 3d narrow scan (**right**).

Figure 15 shows the narrow scan on the Pb 4f_7/2_ peak. The two observed components are attributed to metallic Pb and Pb^2+^, respectively. The B.E. of Pb 4f_7/2_ peak observed at 139.0 eV is consistent with the presence of PbBr_2_ on the surface of the Pb sample, in agreement with previously reported data [69,70]. The Br 3d_5/2_ peak was observed at 68.8 eV. When Br is forming PbBr_2_, a similar Br 3d_5/2_ peak was reported at 68.7 eV [71].

Table 12 shows the experimentally determined B.E. values for the two Pb components and the assigned oxidation states.

The Pb:Br ratio determined from the XPS data is in the range of 35.6:64.4 ≈ 1:1.81, which is close to the expected 1:2 ratio. It should be noted that the occurrence of PbBr_2_ on lead surface was also confirmed by XRD measurements.

The featureless residue obtained with only two components, the relatively narrow Pb^2+^ peak and the rapid decrease of the O1s peak intensity, suggests the absence of any other Pb^2+^ containing compounds. This is in contrast with XRD data, which indicated the occurrence of Pb_3_O_4_ on the sample, along with PbBr_2_. The absence of Pb_3_O_4_ in the XPS spectra could be the result of the different scanning depth and/or different scanning area typical for the two techniques. The metallic component of the Pb 4f_7/2_ peak is probably the effect of sample reduction due to the Ar sputtering.

##### Fe Samples

The elements identified in the XPS spectra for the Fe sample are Fe, O, K, Br and C.

**Figure 16:**
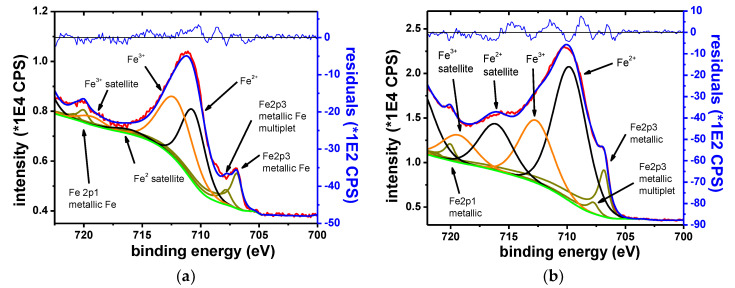
Fe 2p3 narrow scan, initial spectrum (**a**) and spectrum after 15 s of etching (**b**).

Figure 16 shows the Fe 2p_3/2_ narrow scan spectrum, collected on the sample, before (left) and after 15 s of etching (right).

The narrow scans showed that Fe was found in three oxidation states, namely metallic, Fe^2+^ and Fe^3+^. Before etching, the spectra is dominated by the oxide contributions. After etching, the metallic component becomes dominant. The metallic component shows multiplet splitting. The oxide phases show characteristic satellite features. The chemical states are identified according to the B.E. at which they are observed and by their satellite fingerprint. The observed results agree with the previously reported values [44,72,73].

Table 13 shows the B.E. of Fe components and their respective oxidation states.

The O1s peak shows contributions from organic species and inorganic oxides. The two types of contributions can be separated based on their response to the Ar ion etching. The organic contributions vanish more quickly than the contributions associated to the Fe oxides. As expected, the presence of KBr on the metallic surface was also evidenced.

## 4. Conclusions

Based on the obtained results, some conclusions can be drawn:

- Electrochemical measurements showed that the addition of bromine to the system favors to a great extent the dissolution process of all studied metals as compared to bromine-free electrolytes. Better understanding of the reaction mechanisms and evaluating the electrochemical parameters of the systems are necessary before any technological approach. The results of the study concern the dissolution processes, but at the same time they open a real possibility to recover the metals of practical importance from bromine-based systems.
- In the investigated experimental conditions, the highest dissolution rates of the metals were obtained in acidic bromide solution containing 0.01 M Br_2_ and they vary in the following order: Zn >> Sn > Pb > Fe> Cu.
- XPS chemical assessment allowed the identification of the dissolution products formed on the metallic surfaces after exposure to bromine-containing solution in initial stages of the corrosion process. They consist mainly of metallic oxides in the case of Cu, Zn, Sn and Fe samples, while the presence of a layer of PbBr_2_ was noticed on the Pb surface.
- XRD measurements showed that the immersion of the metals in different bromide-based electrolytes induces structural modifications for all electrodes. From the XRD diffractograms obtained on all studied metals, a preferential orientation tendency of the crystallites of the corrosion products was observed according to certain crystallization planes.
- The bromide-based systems are promising alternatives for lixiviants in hydrometallurgical route of metals recovery from WPCBs. Since the major economic driver for WPCBs recycling relies on the efficient metal recovery, in a first step, an effective dissolution of the raw material is necessary. The results of our work show that all studied metals could be successfully leached by using bromine-based systems and indicate good premises for selective and efficient metal recovery through a multi-step hydrometallurgical processing route.

## Figures and Tables

**Table 1:** Corrosion kinetic parameters for metals in different bromide-based electrolytes.

Solution	*E_corr_* (mV vs. Ref.)	*I_corr_* (μA cm^−2^)	$\beta_{a}$ (mV dec^−1^)	$\left|\beta_{c}\right|$ (mV dec^−1^)
**Cu**				
**A**	−316.7	10.7	70.8	879.0
**B**	−321.3	19.5	71.7	428.1
**C**	−251.1	146.2	72.3	198.0
**D**	−279.9	43.4	64.6	-
**Zn**				
**A**	−972.0	8.4	12.6	479.1
**B**	−987.1	26580	145.6	104.9
**C**	−978.2	27960	143.8	123.6
**D**	−971.2	27790	159.3	97.1
**Sn**				
**A**	−409.2	1.4	37.7	79.3
**B**	−503.5	13.4	23.4	187.1
**C**	−483.7	399.1	39.1	148.2
**D**	−481.3	92.2	29.2	164.5
**Pb**				
**A**	−557.9	37.8	32.0	336.2
**B**	−557.9	37.8	32.0	336.2
**C**	−525.9	164.4	24.6	67.9
**D**	−527.3	50.4	25.2	108.3
**Fe**				
**A**	−383.8	2.07	149.8	nd
**B**	−300.2	4.06	27.8	76.0
**C**	−254.2	157.4	23.5	164.5

**Table 2:** Electrochemical parameters of Cu dissolution in different bromide-containing systems.

Sol.	*R_e_* (Ω cm^2^)	*R_ct_* (Ω cm^2^)	*Q_dl_* (μF s^n−1^ cm^−2^)	*n_dl_*	*C_dl_* (μF cm^−2^)	*R_F_* (Ω cm^2^)	*Q_F_* (mF s^n−1^ cm^−2^)	*n_F_*	*C_F_* (mF cm^−2^)	*W* (S s^1/2^ cm^−2^)
**A**	0.79	191.5	9.05	0.91	4.82	3783	0.44	0.46	0.71	-
**B**	0.53	48.78	161.1	0.80	47.93	488.3	1.80	0.51	1.58	-
**C**	0.56	21.54	212	0.79	50.64	38	0.23	0.95	0.18	0.19

**Table 3:** Electrochemical parameters of Zn dissolution in different bromide-containing electrolytes.

Sol.	*R_e_* (Ω cm^2^)	*R_ct_* (Ω cm^2^)	*Q_dl_* (μF s^n−1^ cm^−2^)	*n_dl_*	*C_dl_* (μFcm^−2^)	*R*_1_ (Ω cm^2^)	*Q*_1_ (μF s^n-1^ cm^−2^)	*n*_1_	*C*_1_ (μF cm^−2^)	*R_L_* Ω cm^2^)	*L* (H cm^2^)	*R_2_* (Ω cm^2^)
**A**	1.02	322.9	12.3	0.87	5.5	285.5	89.02	0.70	18.4	71.4	39.8	-
**B**	0.67	0.87	6000	0.55	86.4	-	-	-	-	0.95	0.001	0.065
**C**	0.59	0.29	7200	0.61	141.5	-	-	-	-	0.28	0.0002	0.046

**Table 4:** Electrochemical parameters of Sn dissolution in different bromide-containing solutions.

Sol.	*R_e_* (Ω cm^2^)	*R_f_* (Ω cm^2^)	*Q_f_* (μF s^n−1^ cm^−2^)	*nf*	*C_f_* (μF cm^−2^)	*R_ct_* (Ω cm^2^)	*Q_dl_* (mF s^n−1^ cm^−2^)	*n_dl_*	*C_dl_* (mF cm^−2^)	*R* (Ω cm^2^)	*L* (H cm^2^)
**A**	0.88	61860	34.15	0.91	36.94	41580	0.191	0.85	0.28	-	-
**B**	0.65	0.90	16.84	0.99	16.84	90.13	0.72	0.81	0.39	30.36	18.35
**C**	0.51	0.27	781	0.80	94.1	5.45	6.03	0.79	0.49	11.31	-

**Table 5:** Electrochemical parameters of Pb dissolution in bromide-containing solutions.

Sol.	*R_e_* (Ω cm^2^)	*R_ct_* (Ω cm^2^)	*Q_dl_* (μF s^n−1^ cm^−2^)	*n_dl_*	*C_dl_* (μF cm^−2^)	*R*_1_ (Ω cm^2^)	*Q*_1_ (mF s^n−1^ cm^−2^)	*n*_1_	*C*_1_ (mF cm^−2^)	*W* (S sec^1/2^ cm^−2^)
**A**	0.94	19.63	210.0	0.84	74.16	1096	1.91	0.50	3.9	-
**B**	0.58	0.87	285.0	0.88	91.9	125.7	1.58	0.68	0.25	0.06129
**D**	0.55	0.80	565.0	0.87	170.0	13.39	2.15	0.78	0.79	-

**Table 6:** Electrochemical impedance parameter values for the corrosion of Fe in various bromide-based electrolytes.

Sol.	*R_e_* (Ω cm^2^)	*R_ct_* (Ω cm^2^)	*Q_dl_* μF s^n−1^ cm^−2^)	n_dl_	*C_dl_* (μF cm^−2^)	*R*_1_ (Ω cm^2^)	*Q*_1_ μF s^n−1^ cm^−2^)	n_1_	*C*_1_ (μF cm^−2^)	*R_L_* (Ω cm^2^)	*L* (H cm^2^)
**A**	1.00	42360	116	0.83	155	-	-	-	-	-	-
**B**	0.79	59.60	202	0.77	54.0	1594	48.0	0.91	37.7	-	-
**C**	0.69	9.15	45.1	0.90	18.25	234.7	238	0.70	69.1	116.2	36.91

**Table 7:** Preferential orientation tendency of the crystallites of the corrosion products in different bromide-based electrolytes.

Sample	Relative Intensities Ratio	Sol. A	Sol. B	Sol. C
Cu	I_(111)_/I_(220)_	1.01	1.05	1.10
Zn	I_(002)_/I_(102)_	1.03	1.10	-
Sn	I_(110)_/I_(211)_	0.97	1.01	1.05
Pb	I_(110)_/I_(211)_	0.99	1.01	0.98
Fe	I_(001)_/I_(201)_	1.10	1.12	-

**Table 8:** Cu2p_3/2_ fit parameters (referenced to adventitious carbon C1s peak at 285.1 eV).

Peak	Iteration	B.E. (eV)	FWHM (eV)
Cu 2p_3/2_ Cu (II)	0	934.2	3.1
Cu 2p_3/2_ Cu (I)	9	932.6	2.1

**Table 9:** Cu oxide ratios.

Level	Cu^2+^ (at %)	Cu^1+^/Cu^0^ (at %)
Iteration 0 (“as received” sample)	83.7	16.3
Iteration 9	25.5	74.5

**Table 10:** B.E. and K.E. values for Zn peak contributions/oxidation states.

Peak	Oxidation State	Measured B.E. (eV)	K.E. (eV)	FWHM (eV)
Zn 2p_3/2_	metallic	1021.8	-	1.0
Zn 2p_3/2_	Zn^2+^	1022.5	-	1.4
Zn LMM	metallic	-	991.9	1.2
Zn LMM	Zn^2+^	-	987.5	3.0

**Table 11:** B.E. values for Sn (with reference the C1s peak due to adventitious C which is observed at 285.0 eV).

Peak	Oxidation State	Compound	Measured B.E. (eV)	Reported B.E. (eV)	FWHM (eV)	References
Sn 3d_5/2_ metallic	0	Sn	485.0	485.0	0.6	[68]
Sn 3d_5/2_ oxidized	4+	SnO_2_	486.8	486.6	1.2	[65]

**Table 12:** B.E. values for Pb.

Peak	Oxidation State	B.E. (eV)	FWHM (eV)
Pb 4f_7/2_ metallic	0	137.0	1.0
Pb 4f_7/2_ oxidized	2+	139.0	1.1

**Table 13:** B.E. values for Fe.

Peak	Oxidation State	Measured B.E. (eV)	FWHM (eV)	Reported B.E. (eV)	References
Fe 2p_3/2_ metallic	0	706.8	1.0	706.8	[44,72,73,74]
Fe 2p_3/2_ multiplet	0	707.7	1.0	0.9 (from main peak)	[44,72,73]
Fe 2p_3/2_ (II) oxide	2+	709.8	3.5	709.6–709.9	[44,72,73,75]
Fe 2p_3/2_ (II) satellite	2+	716.1	3.5	-	-
Fe 2p_3/2_ (III) oxide	3+	712.7	3.5	710.8–711.4	[44,72,73,76]
Fe 2p_3/2_ (III) satellite	3+	719.3	3.5	-	-

## Supplementary Materials

The following are available online at https://www.mdpi.com/1996-1944/13/16/3630/s1, Figure S1: Comparison of the experimental impedance data corresponding to the corrosion of different metals in sol. C and the transfer function calculated using K–K relations. Metals: (a) Cu; (b) Zn; (c) Sn; (d) Pb; (e) Fe. Experimental data: (●) Real component (Z’); () Imaginary component (Z”); Calculated points: (■) Real component (Z’); () Imaginary component (Z”), Figure S2: SEM micrographs of the metallic specimens after different immersions times in sol. C: 60 minutes in the case of Cu (a), Sn (b), Zn (c) and 120 minutes for Pb (d) and Fe (e). Inserts: SEM pictures in enlarged scales.
