# Supplementary material for: Dissolution of Metals in Different Bromide-Based Systems: Electrochemical Measurements and Spectroscopic Investigations
Source: Materials (Basel). 2020 Aug 17;13(16):3630. doi: 10.3390/ma13163630 (PMC7475910; doi:10.3390/ma13163630)
Supplement: Supplementary file 1 [file materials-13-03630-s001.pdf]

materials

MDPI

Supplementary materials

Dissolution of Metals in Different Bromide-Based Systems: Electrochemical Measurements and Spectroscopic Investigations

Content included in the supplementary materials:

S-1. Kramers - Kronig transformations of the experimental EIS data

S-2. SEM characterization of the metallic surfaces after exposure to 0.01 M Br_2 – containing electrolyte (sol. C)

S-1. Kramers - Kronig transformations of the experimental EIS data

Figure S1 shows the comparison of the experimental impedance corresponding to different metals corrosion in sol. C and the calculated data using K–K relations.

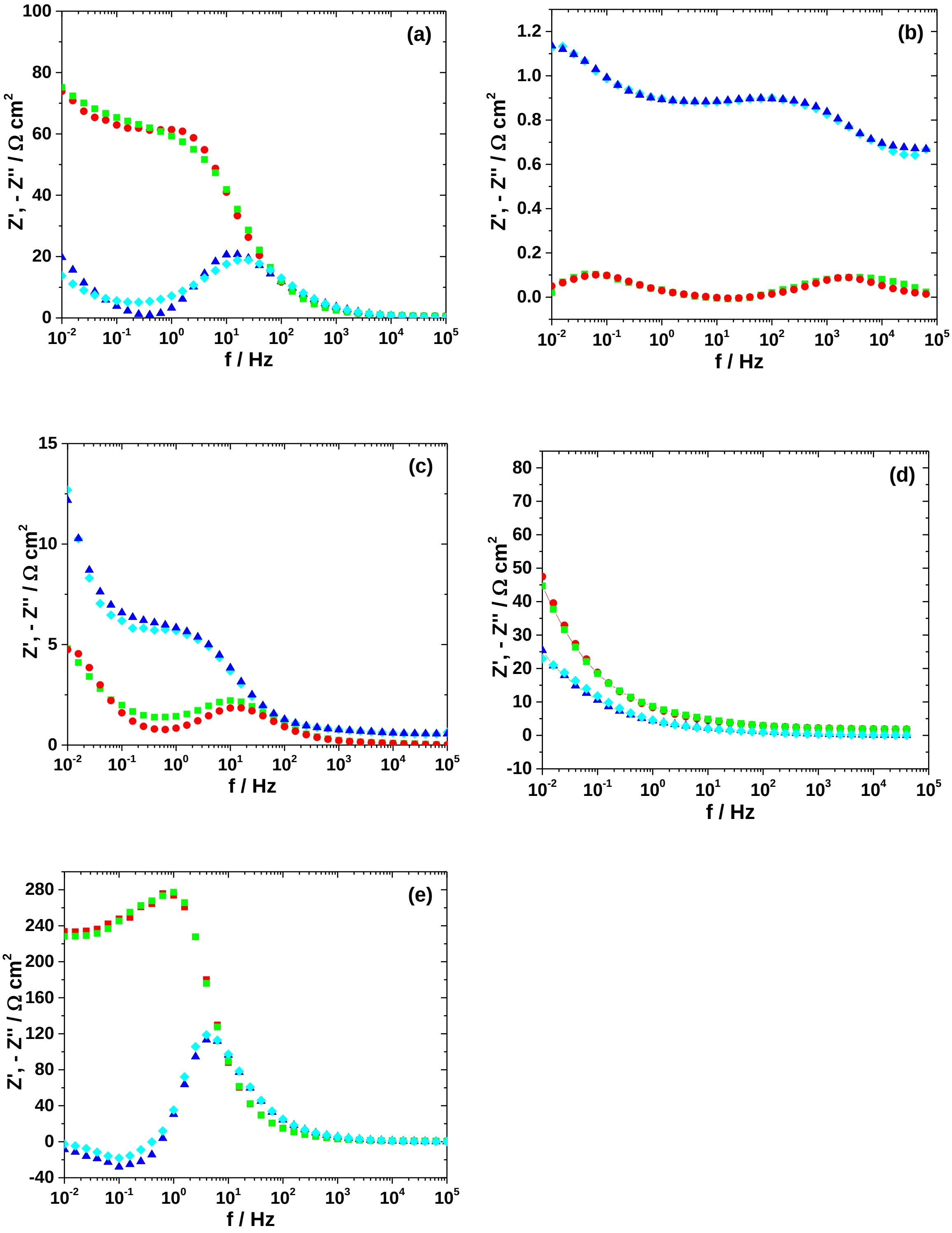

Figure S1. Comparison of the experimental impedance data corresponding to the corrosion of different metals in sol. C and the transfer function calculated using K–K relations. Metals: (**a**) Cu; (**b**) Zn; (**c**) Sn; (**d**) Pb; (**e**) Fe. Experimental data: (●) Real component (Z′); (▲) Imaginary component (Z″); Calculated points: (■) Real component (Z′); (♦) Imaginary component (Z″).

S-2. SEM characterization of the metallic surfaces

In order to examine the surface morphology of the metallic samples after their exposure to 0.01 M Br_2—containing electrolyte (sol. C), SEM images were collected using secondary electrons on a GEMINI 300 SEM (Carl Zeiss, Cambridge, UK) equipment. The results are illustrated in Figure S2.

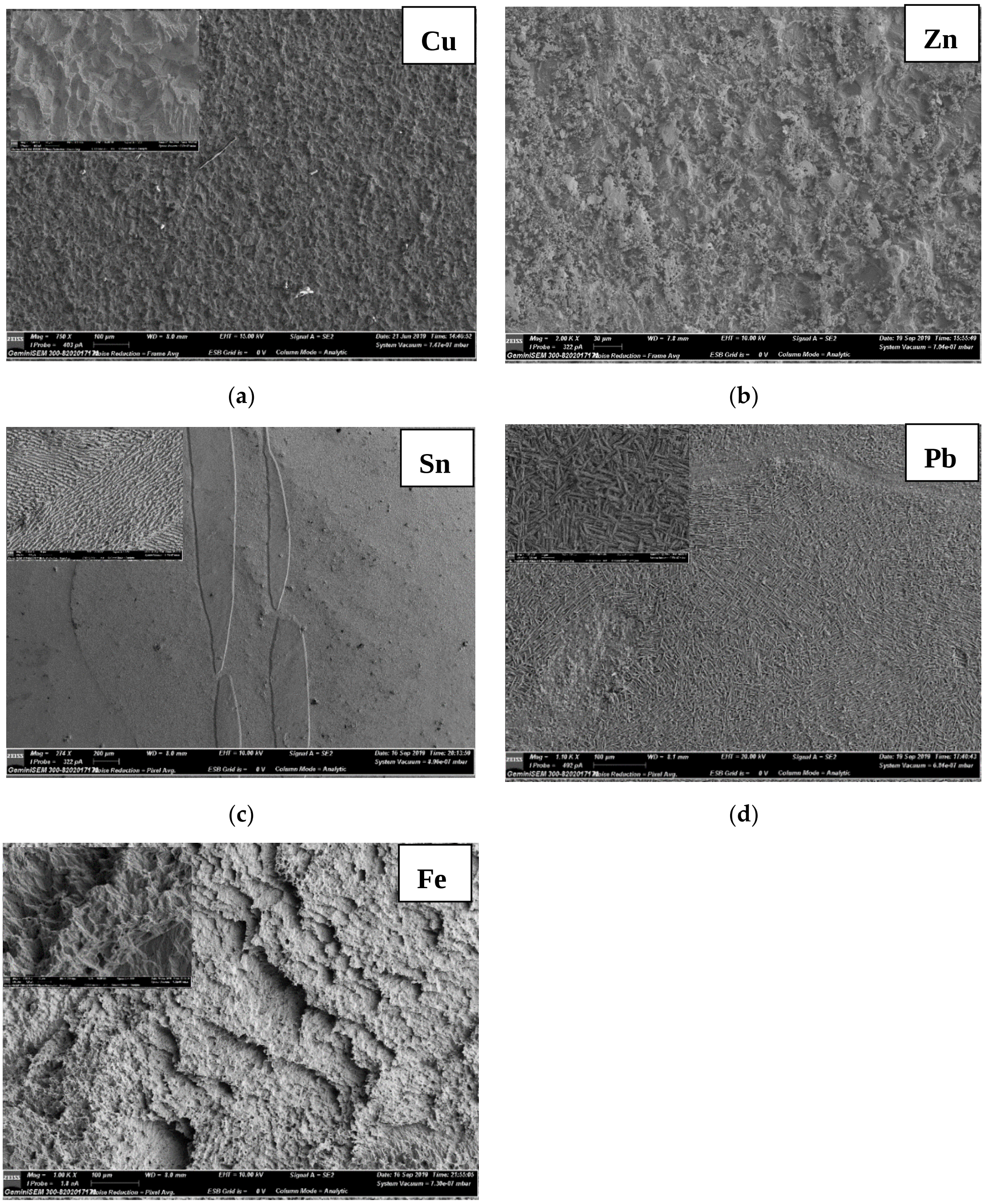

(**a**) (**b**)

(**c**) (**d**)

(**e**)

Figure S2. SEM micrographs of the metallic specimens after different immersions times in sol. C: 60 min in the case of Cu (**a**), Sn (**b**), Zn (**c**) and 120 minutes for Pb (**d**) and Fe (e). Inserts: SEM pictures in enlarged scales.

As shown in Figure S2a, the copper surface is rough and shows clear evidence of metal dissolution. In the case of Zn, the corresponding SEM micrograph (Figure S2b) shows evidence of metal dissolution and some powder material, most probably ZnO deposited on the surface. Although the Sn sample shows a relatively smooth surface, evidences of metal dissolution are still visible in Figure S2c. The Pb electrode presents a layer of well crystallised particles deposited on its surface

(Figure S2d). A very rough surface and clear evidence of the corrosion products could be seen on Fe sample in exposed to Sol. C (Figure S2e).

© 2020 by the authors. Submitted for possible open access publication under the terms and conditions of the Creative Commons Attribution (CC BY) license (http://creativecommons.org/licenses/by/4.0/).
